# Supplementary material for: Co-Occurring Methylenetetrahydrofolate Reductase (MTHFR) rs1801133 and rs1801131 Genotypes as Associative Genetic Modifiers of Clinical Severity in Rett Syndrome
Source: Brain Sci. 2024 Jun 21;14(7):624. doi: 10.3390/brainsci14070624 (PMC11275218; doi:10.3390/brainsci14070624)
Supplement: Supplementary file 1 [file brainsci-14-00624-s001.zip › brainsci-3039386-supplementary.pdf]

Supplementary Information

Supplementary Information S1: Frequency (%) of comorbid diagnoses in the sample (n=65)

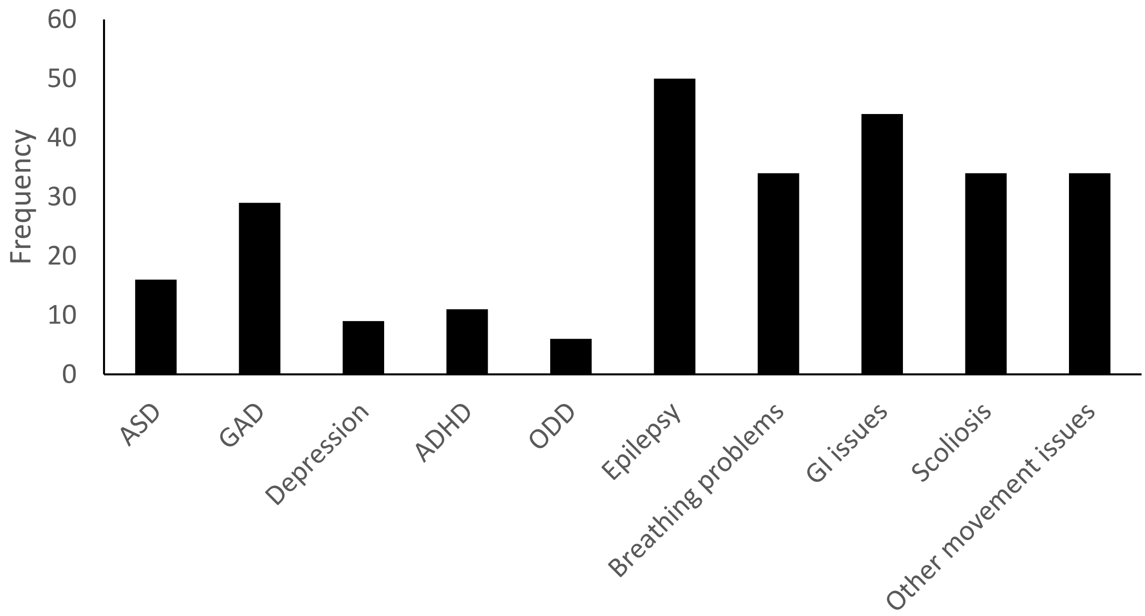

Supplementary Information S2: Frequency (count) of *MTHFR* rs1801133 and rs1801131 genotypes in RTT stratified by age (n=65)

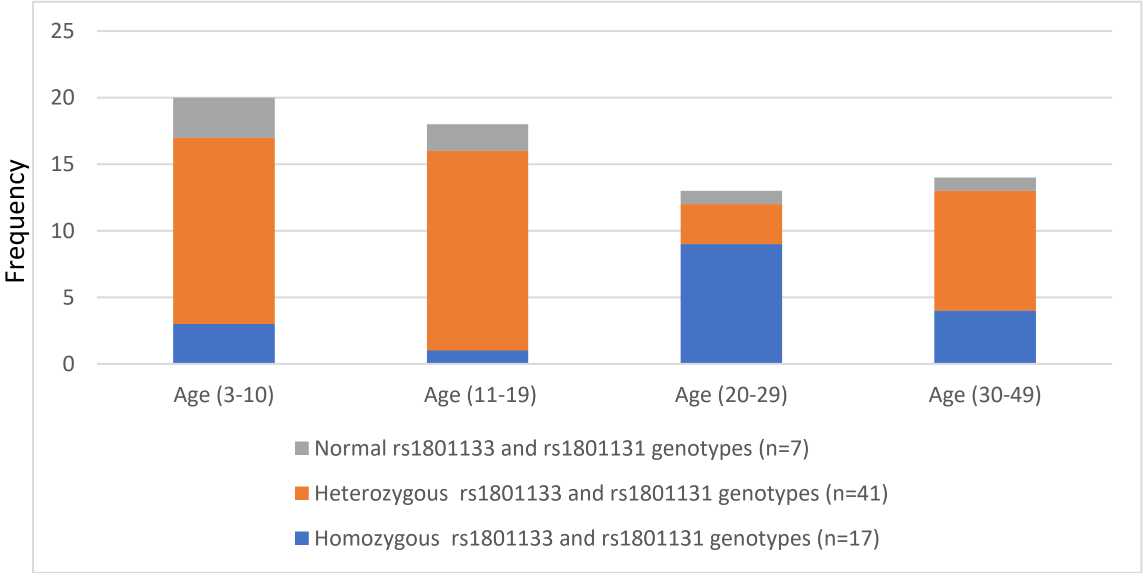

Notes:

Normal: rs1801133 and rs1801131 genotype predicted to have no impact on MTHFR activity as described in the individual pharmacogenomic reports were assigned as ‘normal.’

Heterozygous: rs1801133 and rs1801131 heterozygous genotypes predicted to have either a mild or partial impact upon MTHFR activity as described in the individual pharmacogenomic reports were assigned as ‘heterozygous.’

Homozygous: rs1801133 and rs1801131 homozygous genotypes predicted to have most reduced MTHFR activity as described in the individual pharmacogenomic reports were assigned as ‘homozygous.’

rs1801133 and rs1801131 genotypes for each group are shown in Table 1B.

Supplementary Information S3: Frequency of Different AED use and *MTHFR* rs1801133 and rs1801131 genotypes in RTT

AED*	*MTHFR* rs1801133 and rs1801131 genotypes	
	Homozygous (n=12)	Non-Homozygous (n=33)
Carbamazepine	0	2
Lacosamide	3	2
Lamotrigine	6	12
Levetiracetam	2	8
Oxcarbazepine	0	1
Sodium Valproate	1	6
Topiramate	5	2
Clobazam	0	6
Clonazepam	1	1
Midazolam	0	3
Brivaracetam	0	1
Zonisamide	0	1

Abbreviations: AED (anti-epileptic drugs); MTHFR (methylenetetrahydrofolate reductase)

* AEDs at the time of the PGx test

Supplementary Information S4: Rett Syndrome variants and *MTHFR* rs1801133 and rs1801131 genotypes

**Normal* (n=7)**	**Heterozygous** (n=41)**	**Homozygous*** (n=17)**
Heterozygous for c.473c mutation	Heterozygous for c.422A>C (p.Tyr141Ser) in exon 4 of *MECP2* gene	*MECP2* exon3_4del)
Heterozygous sequence change c.397C>T p.(Arg133Cys) in exon 4 of *MECP2*	*MECP2* mutation -1157del41	c.916C>T - R306C
Genetically confirmed (c.502C>T)	Confirmed genetic *MECP2* mutation	Other *MECP2* mutation or not known
Heterozygous c.1160_*5215del p(Pro387_Ser486delinsGln)	Atypical Genetic diagnosis (*MECP2* missense mutation)	c.808C>T - R270*
Genetically confirmed	Heterozygous for c.473C>T p.(Thr158Met)	Partial deletion of exon 4 of *MECP2* gene
MECP2 variant c.[998_1050del; 1129_1198del; 1245_1249del]	c.916C>T - R306C	*MECP2* deletion C1157_1197del41 - L386fs)
MECP2 mutation positive	Confirmed genetic mutation but not further specified	1150-1153
	Heterozygous c.1160_*5215del p(Pro387_Ser486delinsGln)	Confirmed genetically
	Negative genetic test	*MECP2* Deletion 1164del44
	Other *MECP2* mutation or not known	Heterozygous for *MECP2* c.502C>T
	Molecular confirmation - Deletion on Exons 2 and 4.	MECP2 c.455C>G-P152R
	Heterozygous variant c.799C>T (p.Arg267*) in exon 4 of *MECP2* gene	De novo pathogenic *MECP2* c.905C>A variant
	Atypical - GABBR 2 related disorder	Atypical
	MECP2 positive	P.R270X
	Genetically confirmed	Xq 28 Deletion
	Pathogenic heterozygous deletion of whole exome 3 and portion of exome 4 of *MECP2* gene	Other *MECP2* mutation or not known
	NM_004992.4: c.316C>T p.(Arg106Trp) ChrX:g.153297719G>A	Atypical - Nuclear Ribonucleoprotein U (HNRNPU) mutation
	MECP2 Mutation	
	NM_004992.3:c1164_1207delp(.Pro389Ter}	
	7p14.3 gain variant	
	MECP2 c.808C>T-R280*	
	c.1157_1197del (P.Leu386HisfsTer5)	
	Heterozygous for a deletion of exon 3 and part of exon 4 of *MECP2*	

	Heterozygous for a deletion of part of *MECP2* exon 4	
	c.433C>T Heterozygous	
	Other *MECP2* mutation or not known	
	Other *MECP2* mutation or not known	
	c.880C>T (p.Arg294Ter)	
	Clinical diagnosis	
	MECP2 mutation c.916C>T – R306C)	
	Common *MECP2* point mutation	
	Confirmed deletion of MECP2 gene	
	Other *MECP2* mutation or not known	
	Heterozygous variant *MECP2* gene	
	MECP2 mutation	
	Deletion in *MECP2* gene	
	MECP2 mutation c.8080c>t)	
	Other *MECP2* mutation or not known	
	A heterozygous frameshift mutation c.215delC (p.Pro72Argfs) in exon 3 of the *MECP2* gene.	
	Atypical - Multiple chromosomal abnormalities affecting chromosomes 12 and X	
	c.880C>T; p.Arg294X of MECP2 gene	

Notes: Diagnosis information for Rett Syndrome (RTT) was obtained from either genetic reports or medical information. Where genetic reports were not available the Caregiver was consulted. A clinical diagnosis was confirmed by either a Consultant Child and Adolescent Psychiatrist or an Associate Specialist specializing in RTT alongside previous medical history/case notes.

*Normal: rs1801133 and rs1801131 genotype predicted to have no impact on MTHFR activity as described in the individual pharmacogenomic reports were assigned as 'normal.'

**Heterozygous: rs1801133 and rs1801131 heterozygous genotypes predicted to have either a mild or partial impact upon MTHFR activity as described in the individual pharmacogenomic reports were assigned as 'heterozygous.'

***Homozygous: rs1801133 and rs1801131 homozygous genotypes predicted to have most reduced MTHFR activity as described in the individual pharmacogenomic reports were assigned as 'homozygous.'
